# Improving Individual-Specific SSVEP-BCI with Adaptive Channel and Subspace Selection in TRCA

**DOI:** 10.3390/s26041123

**Published:** 2026-02-09

**Authors:** Hui Li, Guanghua Xu, Shanzheng Feng, Chenghang Du, Chengcheng Han, Jiachen Kuang, Sicong Zhang

**Affiliations:** 1 School of Mechanical Engineering, Xi’an Jiaotong University, Xi’an 710049, China; hueylee@stu.xjtu.edu.cn (H.L.);; 2 Key Laboratory for Manufacturing Systems Engineering, Xi’an Jiaotong University, Xi’an 710049, China; 3 The First Affiliated Hospital, Xi’an Jiaotong University, Xi’an 710049, China; 4 Electronic Materials Research Laboratory, Key Laboratory of the Ministry of Education and International Center for Dielectric Research, School of Electronic Science and Engineering, Xi’an Jiaotong University, Xi’an 710049, China; bearfft@stu.xjtu.edu.cn

**Keywords:** adaptive channel and subspace selection in TRCA (AS-TRCA), electroencephalography (EEG), optimal channel learning and selection (OCLS), optimal subspace selection (OSS), steady-state visual evoked potential (SSVEP)-based brain–computer interface (BCI)

## Abstract

The individual-specific steady-state visual evoked potential (SSVEP)-based brain–computer interface (BCI) is characterized by individual calibration data, resulting in satisfactory performance. However, existing individual-specific SSVEP-BCIs employ generalized channels and task-related subspaces, which seriously limit their potential advantages and lead to suboptimal solutions. In this study, AS-TRCA was proposed to develop a purely individual-specific SSVEP-BCI by fully exploiting individual-specific knowledge. AS-TRCA involves optimal channel learning and selection (OCLS) as well as optimal subspace selection (OSS). OCLS aims to pick the optimal subject-specific channels by employing sparse learning with spatial distance constraints. Meanwhile, OSS adaptively determines the appropriate number of optimal subject-specific task-related subspaces by maximizing profile likelihood. The extensive experimental results demonstrate that AS-TRCA can acquire meaningful channels and determine the proper number of task-related subspaces for each subject compared to traditional methods. Furthermore, combining AS-TRCA with existing advanced calibration-based SSVEP decoding methods, including deep learning methods, to establish a purely individual-specific SSVEP-BCI can further enhance the decoding performance of these methods. Specifically, AS-TRCA improved the average accuracy as follows: TRCA 7.21%, SSCOR 7.61%, TRCA-R 6.58%, msTRCA 7.70%, scTRCA 4.47%, TDCA 2.91%, and bi-SiamCA 3.23%. AS-TRCA is promising for further advancing the performance of SSVEP-BCI and promoting its practical applications.

## 1. Introduction

The brain–computer interface (BCI) represents a promising method for human–computer interaction [1,2], capable of transforming the central nervous system activity into artificial outputs. This enables repair, improvement, replacement, and enhancement of interaction between the natural central nervous system and the internal or external environments [3]. The steady-state visual evoked potential (SSVEP), the most popular non-invasive electroencephalography (EEG)-based BCI paradigm, is a time-locked and phase-locked neural response [4]. SSVEP occurs when presented with a temporally periodic visual stimulus. Neural activity in the primary visual cortex of the occipital lobe is modulated, resulting in a periodic response at the stimulus frequency and its harmonics [5]. SSVEP-BCI involves multiple visual stimulus targets, with each target being a specific command, and the human intention is translated into the command by SSVEP decoding. Undoubtedly, precise decoding is critical for SSVEP-BCI. Individual-specific decoding models can significantly enhance SSVEP-BCI performance. However, constructing individual-specific decoding models with individual-specific channel and task-related subspace selection to further improve performance remains unachieved.

In recent years, the iterative evolution of SSVEP decoding technology has substantially boosted the capability of SSVEP-BCI. Specifically, canonical correlation analysis (CCA) [6] introduced spatial filtering to improve the decoding performance for multi-channel SSVEP signals. To sufficiently utilize the harmonic information, a filter bank CCA (FBCCA) [7] was proposed. Compared to the above calibration-free methods, the calibration-based SSVEP decoding methods achieve satisfactory performance by constructing individual-specific SSVEP-BCI, where SSVEP-BCI is characterized by individual calibration data (or training data, labeled data). Task-related component analysis (TRCA) and the extended version ensemble TRCA (eTRCA) [8] significantly improve SSVEP decoding performance by leveraging calibration data to train spatial filters, which can extract repeatable task-related components across trials. Several studies have attempted to improve the performance of TRCA, such as TRCA-R [9], msTRCA [10], and scTRCA [11]. Additionally, neural networks with powerful nonlinear feature extraction have been successfully applied to SSVEP-BCI, such as convolutional correlation analysis (Conv-CA) [12], Deep Neural Networks (DNNs) [13], bidirectional Siamese correlation analysis (bi- SiamCA) [14], and TRCA-Net [15], demonstrating classification performance close to or even better than TRCA-based methods. More valuable SSVEP features would be beneficial for further improving the performance of these methods.

EEG signals from different subjects exhibit individual-specific characteristics and display varying response patterns to SSVEP. Therefore, they have different optimal channels (electrode locations and numbers) and task-related subspaces. However, the above existing calibration-based methods utilize the same channels, which are generalized channels, and the same subspace corresponding to the largest eigenvalue for all subjects. Consequently, SSVEP-BCIs based on these methods are not purely (or completely) individual-specific SSVEP-BCIs, as they only utilize part of the individual-specific knowledge, leading to suboptimal solutions. This study will fully exploit individual-specific knowledge to establish a purely individual-specific SSVEP-BCI, aiming to improve the decoding performance of existing calibration-based methods such as neural network- and matrix decomposition-based methods.

SSVEP is a localized potential [16], which is generated in the occipital region [6]; picking the optimal channels is critical to boosting the performance of SSVEP-BCI. Channel selection has been widely studied in MI [17], emotion recognition [18], rapid serial visual presentation (RSVP) [19], and epileptic seizure detection [20]. Many optimization schemes such as mutual information [21], Pearson correlation coefficient (PCC) [22], regularization sparsity [23], bispectral-based channel selection (BCS) [24], Harmony Search (HS) [25], and sequential forward floating search (SFFS) [26] and gumbel-softmax for Deep Neural Networks [27] have been proposed. Some SSVEP channel selection methods were presented. PCC [28] was proposed for SSVEP channel selection and has been shown to outperform mutual information, normalized mutual information, and BCS. NeuroXAI [29] integrates the surrogate analysis to optimize EEG channels; the performance seriously relies on the chosen decoding method. In [6], a channel selection method based on CCA was proposed, and the importance of the channel is represented by the CCA coefficients. And eight channels with the largest coefficients are selected to constitute the optimal channel subset. The channels with large absolute values of the spatial filter from CCA weights contribute more to the correlation; ref. [30] selected the channels located in the strongly activated areas (nearing the occipital and parietal lobes), and nine channels (O1, O2, Oz, PO7, PO8, POz, P3, P4 and Pz) were selected. In [7], high classification accuracy was adopted as the criterion for selecting channel locations, and nine channels (Pz, PO5, PO3, POz, PO4, PO6, O1, Oz, and O2) over the parietal and occipital lobes were selected; this channel subset has been widely used in SSVEP decoding algorithm research such as [2,8,9,10,11,31,32,33,34,35,36,37]. Although this channel subset achieves high average recognition accuracy, the fixed nine channels are not optimal for every subject or dataset, which results in suboptimal solutions. Furthermore, directly using classification accuracy as the optimization objective and selecting the optimal channel set by combining different channels is straightforward but incurs significant computational costs. Leveraging subject-specific SSVEP response features instead of classification accuracy offers an effective approach to adaptively select subject-specific channels for each subject. However, this approach has not been thoroughly explored.

Traditional TRCA-based SSVEP decoding methods considered only one task-related subspace that corresponds to the largest eigenvalue [8]; however, more subspaces might be task-related. In [38], a method that combined task consistency test with TRCA-based SSVEP detection was proposed to verify the existence of multiple task-relevant subspaces available for TRCA to improve decoding accuracy. This method can find the optimal numbers of subspaces; however, it requires substantial computational cost and training trials.

In order to tackle the dilemma of the suboptimal solution in the current neural network- and matrix decomposition-based SSVEP decoding methods and further boost the decoding performance of individual-specific SSVEP-BCI, adaptive channel and subspace selection in TRCA (AS-TRCA) was proposed in this study. AS-TRCA involves optimal channel learning and selection (OCLS) and optimal subspace selection (OSS). OCLS picks the optimal subject-specific channels, where a sparse learning method for SSVEP channels with a spatial distance constraint was proposed to optimize valuable channels. OSS adaptively determines the appropriate number of optimal subject-specific task-related subspaces by maximizing profile likelihood. AS-TRCA adaptively learns individual-specific knowledge to establish a purely individual-specific SSVEP-BCI, providing more valuable features for the existing neural network- and matrix decomposition-based SSVEP decoding methods to further improve their performance. The main contributions of this study are as follows:

(1) An adaptive selection strategy named AS-TRCA was proposed to develop a purely individual-specific SSVEP-BCI, further improving the performance of the SSVEP-BCI.
(2) An optimal subject-specific channel optimization and selection method, OCLS, utilizing sparse learning with a spatial distance constraint, was proposed.
(3) An optimal subject-specific task-related subspace determination method, OSS, was proposed.

## 2. Materials and Methods

### 2.1. SSVEP Dataset

Two publicly available and widely used datasets, the Benchmark dataset [35] and the BETA dataset [37], were applied in this study for the study of SSVEP decoding algorithms. The Benchmark dataset collected in the laboratory has a higher SNR; by contrast, the BETA dataset was collected outside the laboratory and is more in line with the characteristics of EEG signals for practical applications. The 64-channel whole-head EEG data were collected during a visual speller task involving 40 flickering stimuli (sinusoidal stimulation; the frequency varied from 8 to 15.8 Hz with a 0.2 Hz interval, and the phases ranged from 0 to 1.5π with a 0.5π interval) by SynAmps2 (Neuroscan Inc.) according to the international 10–20 system. Each trial began with a 0.5 s visual cue and ended with a 0.5 s rest time (before the next trial began). The electrode Cz was used as a reference. The signals were recorded at a sampling frequency of 1000 Hz and subsequently down-sampled to 250 Hz for both datasets. The Benchmark dataset was from 35 healthy subjects (18 males, aged 17–34 years, mean age: 22 years) with normal or corrected-to-normal vision, and each stimulus target was evoked 6 times (6 trials or blocks), while the BETA dataset was collected from 70 healthy subjects (42 males, aged 9–64 years, mean age: 25.14 years) with normal or corrected-to-normal vision, with 4 trials for each target. Within each block, the order of 40 stimuli is randomized. The power-line noise was removed for each trial. More details can be found in [35,37].

In this study, first, the visual system delay corresponds to the data of the first $T_{L}$ ms after stimulus onset was removed, where $T_{L}$ = 140 ms for the Benchmark dataset [35] and 130 ms for the BETA dataset [37]. The EEG data in [$T_{L}$, $T_{L}+T_{w}]$ was utilized for SSVEP decoding, where $T_{w}$ indicates the signal time length used for decoding. Then, for each trial, the SSVEP signal in [*k* × 8 − 2, 90] Hz was obtained by the band-pass filter, where *k* indicates the *k*-th sub-band for performing filter bank analysis [7]. Finally, Z-score normalization was performed on each channel of each trial for all data. No other preprocessing methods were applied.

### 2.2. Task-Related Component Analysis (TRCA)

TRCA, a matrix decomposition-based method, is one of the most widely used calibration-based decoding method for SSVEP due to its superior decoding performance. TRCA leverages calibration data to train spatial filters by maximizing the inter-trial repeatability under the same task conditions, which can extract reliable SSVEP task-related components. In SSVEP-BCI, the data $X_{n,k}\in R^{N_{c}\times N_{s}\;\times {\;N}_{t}\;}$ ($N_{c}$ is the number of channels, $N_{s}$ is the number of sampling points, and ${\;N}_{t}$ is the number of blocks or trials) is the *n*-th class (stimulus target) calibration data from one subject, and the TRCA spatial filters $w_{n,k}\in R^{N_{c}\times 1}$ are designed to maximize inter-trial covariance and can be obtained by solving a Rayleigh–Ritz eigenvalue problem:

(1) $$\underset{w_{n,k}}{\arg max}\frac{w_{n,k}^{T}S_{n,k}w_{n,k}}{w_{n,k}^{T}Q_{n,k}w_{n,k}},$$

where $S_{n,k}\in R^{N_{c}\;\times \;N_{c}\;}$ is an inter-trial cross-covariance matrix and can be expressed as

(2) $$S_{n,k}=\frac{1}{{\;N}_{t}\left({\;N}_{t}-1\right)N_{s}}\sum_{\begin{array}{c} i,j=1\\ i\neq j \end{array}}^{N_{t}}\;cov\left(X_{n,\;\;k}^{\left(i\right)},X_{n,k}^{\left(j\right)}\right),$$

where $X_{n,k}^{\left(j\right)}\in R^{N_{c}\times N_{s}\;}$ is the *j*-th trial in $X_{n,k}$. $Q_{n,k}\in R^{N_{c}\;\times \;N_{c}\;}$ is a covariance matrix that can be obtained by

(3) $$Q_{n,k}=\frac{1}{{\;N}_{t}N_{s}}\sum_{i=1}^{N_{t}}cov\left(X_{n,k}^{\left(i\right)},X_{n,k}^{\left(i\right)}\right).$$

Then, the eigenvector corresponding to the largest eigenvalue of $Q_{n,k}^{-1}S_{n,k}$ is the desired spatial filters $w_{n,k}$. And the individual templates of the *k*-th sub-band of the *n*-th class, $T_{n,k}\in R^{N_{c}\;\times \;N_{s}\;}$, can be obtained by averaging the SSVEP data across all trials:

(4) $$T_{n,k}=\frac{1}{{\;N}_{t}}\sum_{i=1}^{N_{t}}\;\;X_{n,k}^{\left(i\right)}.$$

In the classification task, an ensemble strategy can be employed, and the ensemble spatial filter $W\in R^{N_{c}\times N_{f}\;}$ can be obtained as follows:

(5) $$W=\left[w_{1},w_{2},\ldots ,w_{N_{f}}\right].$$

Finally, the feature of the SSVEP test signal $\tilde{X}\;\in R^{N_{c}\;\times \;N_{s}\;}$ can be extracted by performing Pearson correlation analysis between the test signal $\tilde{X}$ and individual templates $T_{n,k}$:

(6) $$r_{\;n,k}=corr(W^{T}\tilde{X},W^{T}T_{n,k}).$$

When the filter bank strategy is adopted, features from different sub-band components are fused by

(7) $$r_{\;n}=\sum_{k}^{K}w_{k}\cdot r_{n,k}.$$

In the expression, $w_{k}=k^{-a}+b$, and *a* = 1.25, *b* = 0.25 [7]. *k* indicates the *k*-th sub-band. The visual stimulus with the maximum correlation coefficient is considered the final classification result.

### 2.3. Proposed AS-TRCA

Optimal channels (including electrode location and number) vary for different subjects due to individual specificity. However, the existing SSVEP decoding methods use a fixed channel subset (generalized channels), leading to suboptimal solutions and hindering further improvements in the decoding performance of individual-specific SSVEP-BCI. In addition, current TRCA-based SSVEP decoding methods use only one subspace, which results in the waste of task-related features. In this study, adaptive channel and subspace selection in TRCA (AS-TRCA) was proposed to learn and automatically select the optimal subject-specific channels and optimal subject-specific task-related subspaces for each subject for establishing a purely individual-specific SSVEP-BCI. AS-TRCA is divided into three parts: optimal channel learning and selection, optimal subspace selection, and feature extraction and SSVEP recognition via existing methods. It is worth noting that in this study, ‘adaptive’ refers only to cross-subject adaptability to find the optimal subspace and channel for each subject, not to online adaptation. For each subject, after being optimized in the training phase, the channel and subspace will be fixed.

#### 2.3.1. Optimal Channel Learning and Selection (OCLS)

During SSVEP generation and propagation, EEG from scalp recordings is the mixture of SSVEP sources and noise. Spatial filtering can restore SSVEP source signals. Therefore, the absolute value of the spatial filter weight can represent the importance of each channel in SSVEP-BCI [30]. TRCA can obtain the spatial filter, but it cannot achieve channel selection by itself. As the number of task-unrelated channels increases, TRCA may model task-unrelated components, which in turn leads to large biases between the obtained spatial filters and the real spatial filters and performance declines. Inspired by [23], which incorporates CSP with sparse regularization to achieve optimal channel selection for MI, a channel sparsity and selection method OCLS based on incorporating prior knowledge of SSVEP and regularization was proposed for SSVEP. Based on using TRCA to parameterize the channel importance by the spatial filter, OCLS employs a sparsity-promoting regularization term and spatial distance constraint to achieve channel optimization of SSVEP. Specifically, for one subject, the calibration data of all $N_{f}\;$ classes from the first sub-band $X_{n,1}\in R^{N_{c}\times N_{s}\;\times {\;N}_{t}\;}$ are concatenated in the signal length dimension to form the new calibration data $X_{e,1}\in R^{N_{c}\times N_{s}N_{f}\;\times {\;N}_{t}}$, where  n is the *n*-th class calibration data, and e represents all classes used only to distinguish from n. Then, Equation (1) is utilized to train a spatial filter on all classes from calibration data (optimizing a channel subset on all classes for one subject), and it is converted to the following constrained optimization problem:

(8) $$\underset{w_{e,1}}{min}\;w_{e,1}^{T}Q_{e,1}w_{e,1}\;\;s.t.\;\;w_{e,1}^{T}S_{e,1}w_{e,1}=1.$$

The identical solution (the identical local minima) between (8) and the following optimization problem [23,39,40] is as follows:

(9) $$\underset{w_{e,1}}{min}\;w_{e,1}^{T}Q_{e,1}w_{e,1}+\frac{1}{w_{e,1}^{T}S_{e,1}w_{e,1}}.$$

Specifically, Equation (8) can be converted into the following equation by the Lagrange multipliers method and finding the stationary point:

(10) $$Q_{e,1}w_{e,1}=\lambda S_{e,1}w_{e,1}.$$

Similarly, Equation (9) can be converted to

(11) $${\left(w_{e,1}^{T}S_{e,1}w_{e,1}\right)}^{2}Q_{e,1}w_{e,1}=S_{e,1}w_{e,1},$$

${\left(w_{e,1}^{T}S_{e,1}w_{e,1}\right)}^{2}$ can be considered as a nonzero scalar, and any $w_{e,1}$ can always be scaled such as ${\left(w_{e,1}^{T}S_{e,1}w_{e,1}\right)}^{2}=\lambda$; therefore, objective (10) is equivalent to objective (11) (specifically, a solution to (10) is equivalent to the solutions to (11) multiplied by a specific scaling factor). Accordingly, the solutions of (8) and (9) are identical up to an arbitrary scaling factor [23,40]. In channel optimization and selection, it is desirable for unimportant channels to have weights close to zero in $w_{e,1}$, which can be realized by sparsity regularization, a method extensively used in machine learning. Since objective (9) is no longer scale-invariant to $w_{e,1}$, the sparsity regularization term can be directly incorporated to enable sparse solutions. In this study, the $l_{1}$-norm penalty term is utilized and incorporated in (9), which is modeled as follows:

(12) $$\underset{w_{e,1}}{min}\;w_{e,1}^{T}Q_{e,1}w_{e,1}+\frac{1}{w_{e,1}^{T}S_{e,1}w_{e,1}}+\alpha {\left||w_{e,1}|\right|}_{1},$$

where α  is the regularization parameter. To better optimize the channels related to the SSVEP task, the spatial distance constraint was proposed to be incorporated with sparse learning. The principal evoked brain region for SSVEP is the occipital region, where Oz is widely considered to be the most critical SSVEP channel [30]. Due to the brain volume conductor effect, channels closer to Oz may have more SSVEP information. Consequently, this study preserves the more valuable channels through distance constraints in channel sparse learning. The Euclidean distance was used as the basis for the spatial constraints because it is the most direct way of describing the physical distances between channels, capturing the distance relationship to the Oz channel well enough to improve the biological plausibility of the model. Specifically, the spatial distance $D\left(i\right)$ between other channels to Oz is used as the constraint:

(13) $$D\left(i\right)=\sqrt{{\left(x_{i}-x_{Oz}\right)}^{2}+{\left(y_{i}-y_{Oz}\right)}^{2}+{\left(z_{i}-z_{Oz}\right)}^{2}},$$

where ($x_{i},y_{i},\;z_{i})\;$ is the position of the *i*-th channel in Euclidean space, while $\left(x_{Oz},y_{Oz},z_{Oz}\right)$ is the Oz channel. It is worth noting that both datasets provide polar coordinates for all channel positions, which can be directly imported into EEGLAB for analysis. In this study, polar coordinates were converted to Euclidean coordinates using EEGLAB. Since Oz was selected as the anchor point, this inevitably introduced an explicit bias toward selecting Oz. The final optimization objective is

(14) $$\underset{w_{e,1}}{min}\;\;J\left(w_{e,1}\right)≜w_{e,1}^{T}Q_{e,1}w_{e,1}+\frac{1}{w_{e,1}^{T}S_{e,1}w_{e,1}}+\alpha \;{\left\|\left.D⊙w_{e,1}\right\|\right.}_{1},$$

where ⊙ is the Hadamard product (or element-wise multiplication). Since ${\left||w_{e,1}|\right|}_{1}=\sum_{i}^{N_{c}}{\left||w_{e,1}(i)|\right|}_{1}$ is not differentiable everywhere, it is replaced with a twice-differentiable surrogate, and they are sufficiently close to each other:

(15) $${\left||\;w_{e,1}|\right|}_{1}≜\sum_{i}^{N_{c}}\sqrt{{\left||\;w_{e,1}\left(i\right)|\right|}_{2}^{2}+\varepsilon },$$

where ε is a minimal value to prevent the denominator from becoming zero. The derivative of (14) can be computed by

(16) $$\dot{J}\left(w_{e,1}\right)=2Q_{e,1}w_{e,1}-\frac{2S_{e,1}w_{e,1}}{{\left(w_{e,1}^{T}S_{e,1}w_{e,1}\right)}^{2}}+\alpha \left(D⊙J_{1}\left(w_{e,1}\right)\right),$$

where $J_{1}(w_{e,1})$ with the *i*-th element is $\frac{w_{e,1}(i)}{\sqrt{{\left||w_{e,1}(i)|\right|}_{2}^{2}+\varepsilon }}$, which is the derivative of ${\left||w_{e,1}|\right|}_{1}$.

To achieve the objective (14), minFunc based on L-BFGS was utilized in this study [23]. It is a computationally efficient algorithm for unconstrained optimization of differentiable functions. Subsequently, the spatial filter $\hat{w}\;$ after optimization can be obtained.

To realize adaptive optimal channel selection, a channel selection method based on spatial filter weights was proposed. Specifically, this involves selecting the channel whose weight is not significantly lower than the maximum weight to form the optimal channel subset, which can be expressed as

(17) $$Cs=Cs\cup i,\frac{abs\left(\hat{w}\left(i\right)\right)}{max\left(abs\left(\hat{w}\right)\right)}>\beta ,i\in 1,2,\ldots ,N_{c}.$$

Cs is the optimal channel subset, *abs* (*) is the operation of getting the absolute value, and *max* (*) is obtaining the maximum value. β is a constant value to be optimized, serving as a boundary to distinguish the optimal channels based on the relative relationships among spatial filter weights. Algorithm 1 summarizes the whole process of OCLS.
**Algorithm 1** The OCLS method for SSVEP-BCI
**Input**:   Individual calibration data: $X_{e,1}\in R^{N_{c}\times N_{s}N_{f}\;\times {\;N}_{t}\;}$, Regularization parameter: α,    Selection boundary: β,   Maximum number of iterations: max_iteration   Convergence tolerance: *e* **Process**:   Computing $S_{e,1}\;$ by (2) and $Q_{e,1}$ by (3)   Obtaining the initial spatial filter $w_{e,1}$ by TRCA   Computing space distance D by (13)   Constructing optimization objective J by (14)   Constructing derivative $\dot{J}$ by (16)   Sparse learning:
$\;\;\;\;\;\;\;\hat{w}$ ← minFunc (J, $\dot{J}$,$\;w_{e,1}$, max_iteration, *e*)
       Cs ← optimal channel selection by (17)Output:Optimal channel set Cs

Eventually, the channels in Cs are regarded as the new channels, and the entire OCLS operation is repeated until the number of channels no longer decreases to obtain the simplest channel subset. Since the number of channels is either decreasing or remaining constant, this process converges monotonically.

#### 2.3.2. Optimal Subspace Selection (OSS)

In TRCA-based methods, the performance of SSVEP decoding can be improved by using multiple task-related subspaces; however, only one subspace is employed in traditional TRCA-based approaches, which results in a waste of valuable features. Here, optimal subspace selection (OSS) was proposed to adaptively determine the appropriate number of subspaces, which are all task-related. Inspired by [41,42], which employed profile log likelihood to automatically select the desired dimension from high-dimensional space, OSS employed profile likelihood to automatically pick the desired task-related subspaces. The eigenvalues λ obtained from TRCA characterize inter-trial task consistency [8,38,43], and the eigenvalues corresponding to the task-related subspace are significantly larger than the task-unrelated ones. Accordingly, it is desirable to pick the subspaces corresponding to the significantly larger eigenvalues as the optimal subspaces. The profile log likelihood can find a ‘big gap’ by maximizing the profile likelihood function [41], effectively splitting the significantly large elements. The profile likelihood method is effective in avoiding falling into local optimal solutions by globally optimizing the profile likelihood function, rather than relying on local gradient information. Therefore, it can be employed to obtain the desired eigenvalues. Specifically, $\lambda_{i}$ ∈ {$\lambda_{1}$, $\lambda_{2}$, …, $\lambda_{M}$}, $\lambda_{1}$ > $\lambda_{2}$> …> $\lambda_{M}$ are the eigenvalues from ${\hat{X}}_{e,1}\in R^{N_{c1}\times N_{s}N_{f}\times N_{t}}$ by TRCA, where ${\hat{X}}_{e,1}\subseteq$ $X_{e,1}$, $N_{c1}$ is the channel number of optimal channel subset Cs after OCLS, and *M* ≤ $N_{c1}$ (in general *M* = $N_{c1}$). The eigenvalues of TRCA are split into two groups based on the number m ∈ {1, 2, …, *M*}, and the corresponding mean and variance can be calculated by

(18) $$\mu_{1}\left(m\right)=\frac{\sum_{i=1}^{m}\lambda_{i}}{m},\mu_{2}\left(n\right)=\frac{\sum_{i=m+1}^{M}\lambda_{i}}{M-m},$$

(19) $$\sigma^{2}\left(m\right)=\frac{\sum_{i=1}^{m}{\left(\lambda_{i}-\mu_{1}\left(m\right)\right)}^{2}+\sum_{i=m+1}^{M}{\left(\lambda_{i}-\mu_{2}\left(m\right)\right)}^{2}}{M},$$

Then, the profile likelihood l(m) can be obtained by

(20) $$l\left(m\right)=\sum_{i=1}^{m}N\left(\lambda_{i}|\mu_{1}\left(m\right),\sigma^{2}\left(m\right)\right)+\sum_{i=m+1}^{M}N\left(\lambda_{i}|\mu_{2}\left(m\right),\sigma^{2}\left(m\right)\right).$$

Finally, the index $N_{sp}$ corresponding to the maximum profile likelihood is the number of optimal task-related subspaces. Algorithm 2 summarizes the whole process of OSS.

**Algorithm 2** The OSS method for SSVEP-BCI**Input**:   Individual calibration data with subject-specific channels: ${\hat{X}}_{e,1}\in R^{N_{c1}\times N_{s}N_{f}\;\times {\;N}_{t}\;}$ **Process**: Obtaining eigenvalues by TRCA and descending sort $\lambda_{1}$ > $\lambda_{2}$> …> $\lambda_{M}$   **For**  m ← 1 to *M*:     **Do** compute $\mu_{1}\left(m\right)$
$,\;\;\mu_{2}\left(m\right)\;$
$\;\mathrm{by}\;(18)\;\mathrm{and}\;\sigma^{2}\left(m\right)$ by (19)
       compute 
$l\left(m\right)$ by (20)
$N_{sp}$
$\;\;\mathrm{index}(\max\{\;l\left(m\right)$})**Output**: the number of optimal task-related subspaces
$N_{sp}$

#### 2.3.3. Feature Extraction and SSVEP Recognition

After applying OCLS and OSS, subject-specific channels and subject-specific task-related subspaces are obtained. These can then be used to train a decoding model based on existing neural network- and matrix decomposition-based SSVEP decoding methods. This study, however, focuses solely on demonstrating the process using TRCA; the only difference is the additional subspace feature fusion process.

Individual calibration data with subject-specific channels is used to train spatial filters on each class, where the spatial filters corresponding to the largest $N_{sp}$ eigenvalues are retained for feature extraction. Similar to the filter bank strategy, features from multiple subspaces will be weighted and fused by

(21) $$r_{\;n}=\sum_{i}^{N_{sp}}\rho_{i}\cdot r_{\;n,i},$$

where, $r_{\;n,i}$ is the feature extracted by the *i*-th task-related subspace (or spatial filter) of the *n*-th class, which is similar to (6). $\rho_{i}$ is the weight of the *i*-th subspace, which can be calculated by

(22) $$\rho_{i}=exp\left(-\frac{i}{2\gamma^{2}}\right),$$

where γ is a constant value to be optimized. The visual stimulus with the maximum correlation coefficient max{$r_{\;n}\}$ is treated as the final classification result. Algorithm 3 summarizes the whole process of AS-TRCA.
**Algorithm 3** The AS-TRCA method for SSVEP-BCI
**Input**:   Individual calibration data: $X\in R^{N_{c}\times N_{s}\;\times {\;N}_{t}\;\times \;N_{f}}$   Test data: x $\in R^{N_{c}\times N_{s}\;}$ **Process**:   Obtaining: $X_{e,1}\in R^{N_{c}\times N_{s}N_{f}\;\times {\;N}_{t}\;}$   Initialization: old_$N_{c}$ ← $N_{c}$   **While** (1)      
Cs ← OCLS ($X_{e,1}$)       $N_{c1}$ ← length  (Cs)         **If**   $N_{c1}$ == old_$N_{c}$
                **break**         **else**               old_$N_{c}$ ←$N_{c1}$  Obtaining: ${\hat{X}}_{e,1}\in R^{N_{c1}\times N_{s}N_{f}\;\times {\;N}_{t}\;}$

${\;\;N}_{sp}$ ← OSS (${\hat{X}}_{e,1}$)   TRCA or other methods:      Feature extraction and Subspace feature fusion by (21)      Classification**Output**: Classification result


### 2.4. Evaluation

#### 2.4.1. Compared Methods

To evaluate the performance advancements of AS-TRCA, comparisons were made with SSVEP channel selection methods, including TRCA_W [30], Binary Harmony Search (BHS) [25], and NeuroXAI [29], as well as the task-related subspace selection method TRCA_TCT [38] and the combination of TRCA_W and TRCA_TCT. In TRCA_W, the channels with the largest 9 absolute values of the spatial filter from TRCA based on all 64 channels were selected as the optimal channel subset for one subject, while NeuroXAI selected the channels with the largest 9 importance weights; other settings were the same as in the original study. All channel selection methods employed all 64 channels as the initial channel set, the same as AS-TRCA. And the channel selection was applied using training data only within each fold. And the test data employed the channel set optimized from the training set. The optimized channel set may vary for each subject across different folds. In this study, only the optimal channels for the current fold were used for each fold. Similar to AS-TRCA, after channel optimization, these methods employ TRCA for feature extraction and classification, with all other hyperparameters consistent with the OCLS settings in AS-TCA.

Additionally, AS-TRCA improves the decoding performance for SSVEP solely by employing individual-specific knowledge (individual-specific channels and task-related subspaces). It does not directly enhance the TRCA feature extraction capability. Therefore, it is reasonable to use AS-TRCA as a new strategy to validate its potential for boosting the performance of other SSVEP decoding algorithms. Therefore, the performance of purely individual-specific SSVEP-BCI built by AS-TRCA combined with the existing method will be evaluated, and the scTRCA [11], msTRCA [10], SSCOR [36], TRCA-R [9], TDCA [33], and neural network method bi-SiamCA [14] are integrated with AS-TRCA separately to investigate the performance gains. Their specific configurations were set to match those in the original studies. For the filter bank strategy (FB-), the number of sub-bands was set to 5 for both datasets [35,37]. And the original channels are 9 channels (Pz, PO5, PO3, POz, PO4, PO6, O1, Oz, and O2) widely used in SSVEP decoding algorithm research. And the bi-SiamCA only employs OCLS and does not use OSS.

#### 2.4.2. Data Analysis

The leave-one (block)-out cross validation was used, where each block served as test samples once. The averaged classification accuracy across all subjects was used to compare the performance among different methods. The error bars indicate standard deviations. The significant difference between the proposed method and the compared method was tested by a paired *t*-test (the Holm–Bonferroni correction was applied) in this study.

## 3. Results

**Figure 1:**
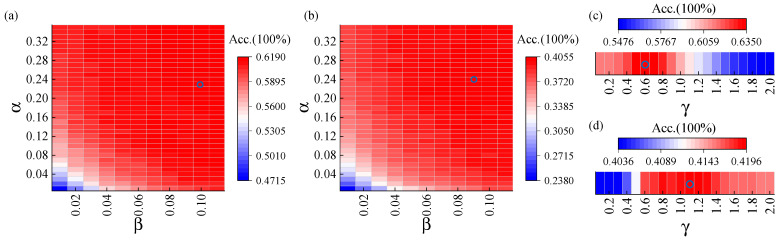
Maximum classification accuracy in the grid search at signal length 0.5 s: (**a**,**c**) show the results of benchmark dataset, while (**b**,**d**) present the results of BETA dataset. (**a**,**b**) correspond to channel selection parameter optimization, while (**c**,**d**) show subspace feature fusion parameter optimization. The location of blue circle indicates the maximum classification accuracy.

The grid search involved optimizing α, β, γ to maximize the classification accuracy, and leave-one (block)-out cross validation was used. Since subspace feature fusion and channel optimization are independent of each other, α and β were optimized simultaneously using the 64-channel data, and γ was optimized in a separate study using subject-specific channels. α was determined from {0.01, 0.02, …, 0.35}, β from {0.01, 0.02, …, 0.11}, and γ from {0.1, 0.2, …, 2}. The results are shown in Figure 1. Overall, α, β, γ maintain superior performance across a wide range. Specifically, OCLS achieves better classification performance when α is in the range of [0.11, 0.35] (this can be considered as a candidate optimal range) for the benchmark dataset and [0.15, 0.35] for the BETA dataset, and β in the range [0.04, 0.35] for both datasets. OSS performs better when γ is in the range [0.5, 0.8] for the benchmark dataset and [0.6, 2] for the BETA dataset. However, this study did not choose the optimal parameters with the highest classification accuracy for each dataset in order to ensure the generalization ability of the parameters and to avoid overly optimistic results. Instead, the same parameters, α= 0.18, β=0.05, and γ=0.7, were randomly determined in the candidate optimal ranges for both datasets. And max_iteration = 1000, $e=10^{-9}$, and $\varepsilon =10^{-10}$.

### 3.1. Comparison of the Classification Performance

To evaluate the classification performance and effectiveness of AS-TRCA, several SSVEP channel selection methods were employed for comparison. These included TRCA_W, BHS, and NeuroXAI and were compared with the proposed OCLS in AS-TRCA. Additionally, the task-related subspace selection method TRCA_TCT was compared with the proposed OSS in AS-TRCA. Furthermore, standard TRCA with original channels and the combination method of TRCA_W and TRCA_TCT (Comb) were also compared with AS-TRCA. AS-TRCA utilized the 64-channel approach to acquire subject-specific channels and task-related subspaces.

Table 1 shows the averaged accuracy across all signal lengths and all subjects, revealing that the proposed individual-specific channel selection method OCLS and the task-related subspace selection method OSS remarkably outperform compared methods (all *p* < 0.001), while AS-TRCA outperforms standard TRCA and Comb (all *p* < 0.001). AS-TRCA is capable of adaptively selecting the optimal individual-specific channels and subspaces for each subject. For computational time cost, although AS-TRCA indeed requires a longer training time than TRCA and TRCA_W, this is deemed acceptable in practical application.

Table 2 shows the averaged classification performance of AS-TRCA as an adaptive selection strategy (AS) combined with existing individual-specific decoding methods, where AS obtains optimal individual-specific channels and subspaces for these methods. The results reveal that the combination of AS-TRCA and these methods significantly increases the classification performance of SSCOR, TRCA, TRCA_R, msTRCA, scTRCA, TDCA, and bi-SiamCA (all *p* < 0.001 for both datasets); in other words, the purely individual-specific SSVEP-BCIs developed by AS-TRCA outperform the existing individual-specific SSVEP-BCIs. Moreover, AS-TRCA is generally applicable, and it can be integrated with almost all SSVEP calibration-based decoding methods, especially matrix decomposition and deep learning methods.

### 3.2. Comparison of Performance with Different Strategies

The ensemble spatial filter strategy (such as eTRCA) and filter bank strategy (such as FBCCA and FB-TRCA) have tremendous achievements in SSVEP-BCI classification tasks. AS-TRCA as a new strategy (adaptive selection strategy, AS) selects individual-specific knowledge (channels and subspaces). Therefore, the performance of AS-TRCA will be compared with these two strategies, and the effect of combining AS-TRCA with these two strategies will also be investigated in this section. TRCA with standard (standard TRCA), FB- (TRCA with the bank strategy, FBTRCA), e (TRCA with the ensemble spatial filter strategy, eTRCA), and FB-e (TRCA with the filter bank and ensemble spatial filter strategy, FB-eTRCA) are traditional methods employing original channels and one task-related subspace, while AS-TRCA with standard (standard AS-TRCA), FB- (AS-FBTRCA), e (AS-eTRCA), and FB-e (AS-FB-eTRCA) indicate using AS with 64 channels. Compared to TRCA and FB-TRCA, eTRCA and FB-eTRCA demonstrate stronger robustness with respect to the number of channels, allowing more channels to enhance decoding performance. Therefore, β was set to 0.04 to acquire more channels for AS-eTRCA and AS-FB-eTRCA, which is smaller than β=0.05 for TRCA and FB-TRCA w/AS.

**Figure 2:**
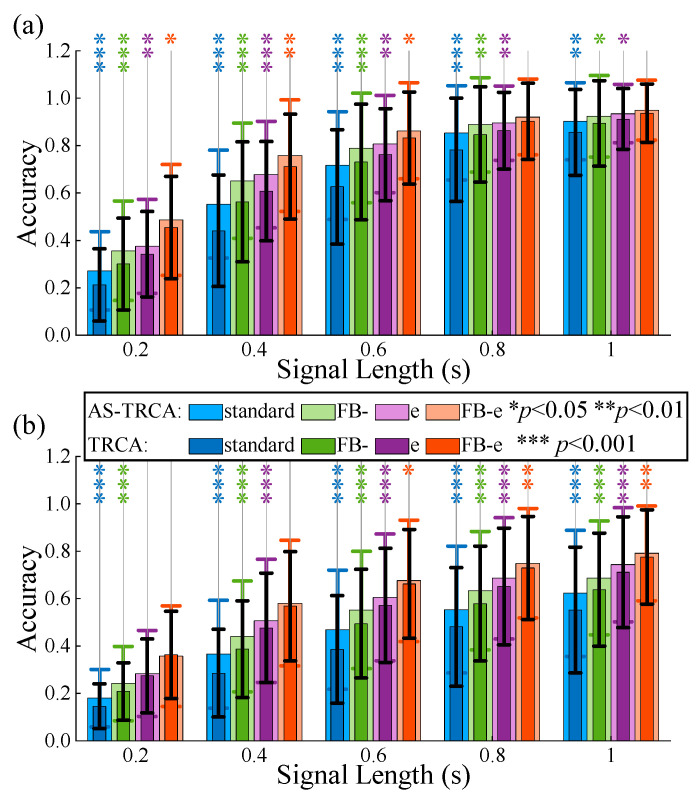
Comparison of performance with different strategies. (**a**) is the benchmark dataset. (**b**) is the BETA dataset. The standard is without any strategy, e is the ensemble spatial filter strategy, FB- is the filter bank strategy, and FB-e refers to employing these two strategies.

Figure 2 shows the averaged results across all subjects. The classification performance of AS-TRCA is inferior to FB-TRCA, eTRCA, and FB-eTRCA. However, AS-TRCA significantly enhances their performance; in other words, the adaptive selection strategy will facilitate the ensemble spatial filter strategy and filter bank strategy to further improve the decoding performance by constructing a purely individual-specific SSVEP-BCI.

### 3.3. Effect of the Number of Calibration Trials

**Figure 3:**
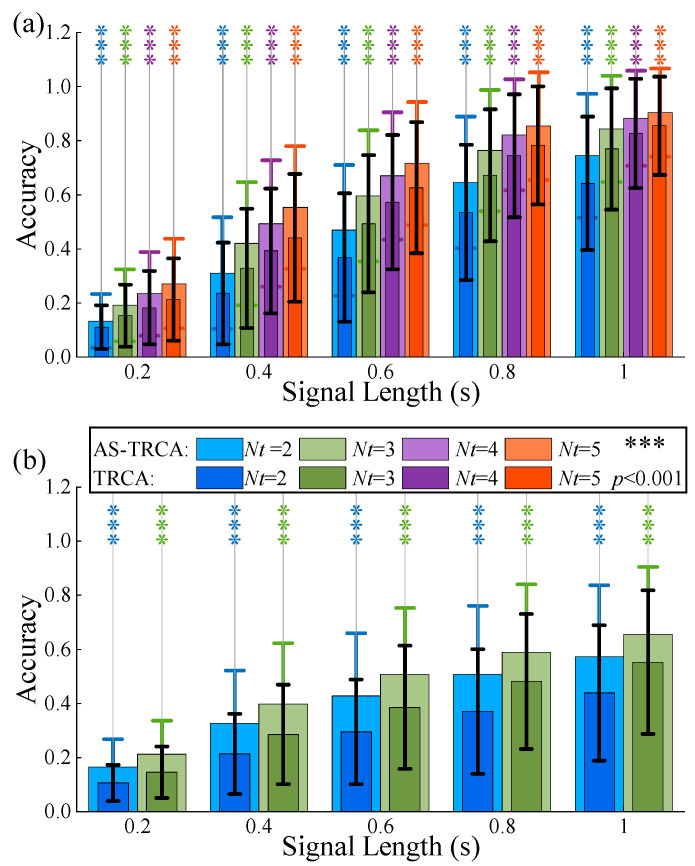
The effect of the number of calibration trials. (**a**) is the benchmark dataset. (**b**) is the BETA dataset.

The number of calibration trials is a critical parameter for maintaining the decoding performance of TRCA. In this section, the effect of the number of calibration trials on AS-TRCA is investigated. The leave-${\;N}_{t}$-out strategy was used; each trial set from $C_{{\;N}_{all}}^{{\;N}_{t}}$ was used to train TRCA for each number of calibration trials, and all remaining trials served as test data. ${\;N}_{t}$ values were set to 2, 3, 4, and 5 in the Benchmark dataset, while 2 and 3 were set in the BETA dataset. ${\;N}_{all}\;=6$ in the Benchmark dataset, and 4 in BETA. The averaged accuracy of all test trials of all subjects for each number of calibration trials was calculated, as shown in Figure 3. The effect of the number of calibration trials on AS-TRCA is consistent with the effect on TRCA; classification accuracy increased with the number of trials. In addition, AS-TRCA dramatically outperformed TRCA under the conditions of different numbers of calibration trials and different lengths for both datasets (all *p* < 0.001).

### 3.4. Effect of the Number of Channels

**Figure 4:**
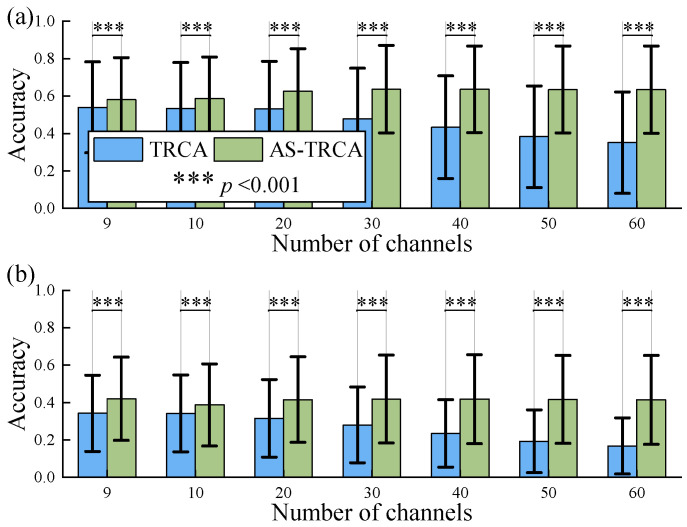
The effect of the number of channels. (**a**) is the benchmark dataset. (**b**) is the BETA dataset.

In order to explore the effect of the number of channels on AS-TRCA and to verify the robustness of the algorithm regarding channels, the numbers of channels were set to 9 (original channels), 10, 20, 30, 40, 50, and 60, respectively. When the number of channels exceeds 9, the newly added channels are the closest to the Oz channel among the remaining channels. The signal length is 0.5 s. The averaged accuracy is shown in Figure 4. It can be observed that the classification performance of TRCA decreases with an increasing number of channels, while AS-TRCA increases and then stabilizes. The effect of the number of channels was assessed by analyzing the standard deviation and range of accuracy across different numbers of channels. The standard deviations are AS-TRCA 2.42% vs. TRCA 7.71% in the Benchmark dataset and 1.15% vs. 7.08% in the BETA dataset. The ranges are AS-TRCA 5.48% vs. TRCA 18.80% in the Benchmark dataset, and 3.34% vs. 17.38% in the Beta dataset. As a result, AS-TRCA has stronger robustness to the number of channels compared to TRCA.

### 3.5. Ablation Study

In order to expose the contribution of each component and to diminish the redundancy in AS-TRCA, ablation studies were conducted and are presented in this section. Table 3 shows the results of the ablation study at the signal length of 0.5 s with all 64 channels. The recognition accuracy of the standard TRCA with original channels as the baseline is marked below the dataset name. Case (a) represents the absence of optimal channel learning and selection (OCLS), utilizing the original channels. Case (b) represents OCLS without spatial distance constraints. Case (c) indicates that optimal subspace selection (OSS) is not considered. Case (d) is the complete AS-TRCA architecture. From cases (a), (c), and (d), it can be found that both OCLS and OSS proposed in this study are conducive to improving the performance of TRCA. From cases (a) and (b), OCLS without spatial distance constraints performs poorly under existing conditions. From cases (a), (b), and (d), it becomes evident that the spatial distance constraint plays a pivotal role in optimizing subject-specific channels in OCpLS. From cases (a) and (c), it is obvious that compared to subject-specific task-related subspaces, subject-specific optimal channels demonstrate greater potential in providing discriminable characteristics for classification tasks. These results collectively underscore the indispensable nature of OCLS, OSS, and spatial distance constraints in enhancing the performance of AS-TRCA.

## 4. Discussion

**Figure 5:**
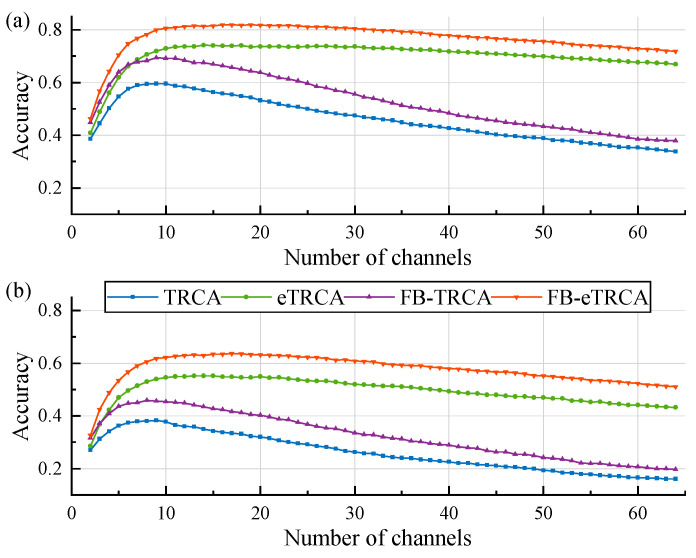
The effect of the number of channels for TRCA with different strategies. (**a**) is the benchmark dataset. (**b**) is the BETA dataset.

Selecting appropriate channels is essential for EEG-BCI. Multi-channel signals are utilized in SSVEP-BCI to improve the SNR. Selecting a small number of channels results in a waste of channel information, while too many channels, aside from demanding high computational costs, introduce excessive noise, potentially leading to the modeling of noise or overfitting. These are not conducive to achieving high-performance SSVEP-BCI. Figure 5 illustrates the impact of the number of channels on TRCA, FB-TRCA, eTRCA, and FB-eTRCA. The channels with the largest absolute weights of the spatial filter from TRCA were selected. The results indicate that all four methods initially exhibit an increase followed by a decrease with the number of channels. Additionally, while activity in the primary visual cortex (V1) is often associated with the generation of SSVEP signals, the dominant sources remain uncertain. Besides the occipital lobe, EEG signals from other regions also significantly contribute to enhancing SSVEP decoding, such as the parietal lobe. These reveal that picking the correct channels is a challenge.

The essence of traditional channel selection methods is to search for the optimal channel set by traversing different channel subsets such as forward selection methods, Harmony Search [25], and NeuroXAI [29], but AS-TRCA obtains the optimal channels through sparse learning of channels based on spatial distance constraints with the optimization goal of maximizing the repeatability of the task-relevant components of SSVEP, which is grounded in the generation mechanism and propagation of SSVEP. SSVEP exhibits time- and phase-locked characteristics and is repeatable across multiple trials, whereas noise is not. As a result, AS-TRCA can achieve a better representation of important channels. Compared to traditional regularization methods, AS-TRCA not only incorporates SSVEP prior knowledge, but also implements a dual-objective optimization architecture, which has the optimization objective of simultaneously maximizing the importance of the channels (via regularization) and minimizing the spatial distances between the channels (via the spatial distance constraints), resulting in a task-relevant, more consistent, and more reliable channel selection result. Experimental results demonstrate the significant advantages of AS-TRCA in terms of computational efficiency and performance.

**Figure 6:**
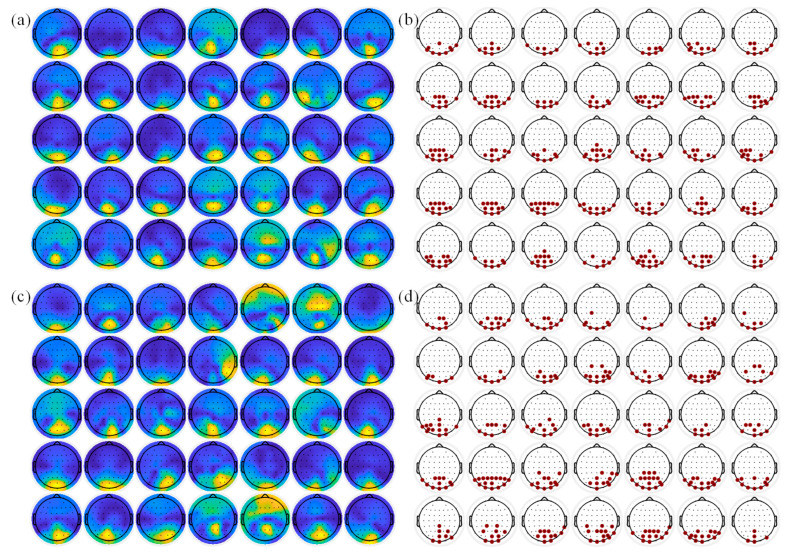
The visualization of selected channels by AS-TRCA and the induced spatial patterns of SSVEP. (**a**,**b**) are spatial patterns and the selected individual-specific channels of the 35 subjects for the Benchmark dataset, respectively, while (**c**,**d**) are the corresponding results for the BETA dataset.

**Figure 7:**
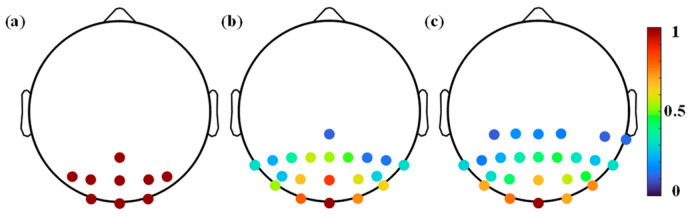
Comparison between the optimal channels selected by ASTRCA and the original channels across all subjects. (**a**) shows the original channels. (**b**) shows the optimal channels from AS-TRCA in the Benchmark dataset, while (**c**) is from the BETA dataset. Different colors indicate the probability that the channel is selected among all subjects.

Quantifying the importance or contribution of a channel in a multi-channel signal is the difficulty in SSVEP channel selection. Currently, channel importance is mainly assessed by classification accuracy [7,44] and spatial filter weights [30], and their effectiveness has been validated in previous studies. Compared to spatial filter weights, the classification accuracy metric is easy to achieve but requires more training data and substantial computation time. Therefore, spatial filter weight was employed in this study. Then, adaptively determining the number of optimal channels is critical. Obviously, a simple thresholding method is not capable of this task. In this study, the channels are adaptively determined based on the relative relationship between their weights, with the channels showing relatively dominant spatial filter weights being selected. As can be seen from Figure 6, the spatial patterns of SSVEPs induced by different subjects show significant differences, and AS-TRCA can adaptively select subject-specific channels. Figure 7 illustrates the comparison between the optimal channels selected by AS-TRCA and the original channels. Different colors indicate the probability that the channel is selected among all subjects. To avoid small-probability events on individual subjects, channels with a selection probability lower than 10% were not displayed. Compared to the fixed original channels, the optimal channels from AS-TRCA seriously deviate from the original channel configuration, and AS-TRCA would acquire valuable channels in a wider range of brain regions. Even though other brain regions also have SSVEP useful information, the main contribution comes from the occipital region. The different selection probabilities of the channels also indicate the significant discrepancy in the optimal channels among subjects. From Figure 7b,c, it can be observed that the selected optimal channels for the subjects may vary in different environments. Incorporating the results of the optimal channel number in Table 1, AS-TRCA tends to select more channels for more information when SSVEPs have higher noise.

In TRCA-based methods, the decoding performance for SSVEP can be improved by utilizing multiple task-related subspaces. The challenge lies in distinguishing these task-related subspaces effectively. In [38,43], the task consistency test was used to pick the task-related subspaces, and the process is complex and requires thousands of TRCA operations to derive the null distribution of the eigenvalues. The task-related subspace is characterized by larger eigenvalues compared to the task-unrelated eigenvalues. The profile likelihood enables the identification of the first several eigenvalues that exhibit a substantial difference from the remaining ones in a descending series (looking for a ‘big gap’ or an ‘elbow’ in this series). This approach aligns well with the requirement for selecting task-related subspaces. Consequently, in this study, the process of selecting the appropriate number of task subspaces was simplified to maximize the profile likelihood of the eigenvalues. The results in Section 3.1 demonstrate that the OSS dramatically outperforms the task consistency test.

Although AS-TRCA further improves the performance of SSVEP-BCI, it still has the following shortcomings: First, the performance of AS-TRCA is limited by the amount of calibration data; as shown in Figure 3, the larger the amount of calibration data, the better the performance of AS-TRCA. Second, the subspace and channels of AS-TRCA will be fixed after optimization, which is not conducive to enabling SSVEP-BCI to face complex and variable human states and environments and highlights the need for the study of an online adaptive mechanism, which allows the AS-TRCA to evolve continuously over time. Third, the performance of AS-TRCA may be sensitive to preprocessing choices, electrode montage geometry, and different hardware and recording conditions, which are well worth further study. These will be the key research directions in the future.

## 5. Conclusions

This study introduces AS-TRCA, an adaptive selection strategy aimed at developing purely individual-specific SSVEP-BCI by acquiring optimal subject-specific channels and task-related subspaces. Specifically, an optimal channel learning and selection method, OCLS, is proposed, which acquires the optimal subject-specific channels through sparse learning with spatial distance constraints. Additionally, an optimal subspace selection method OSS is introduced, which adopts profile likelihood to adaptively determine optimal subject-specific task-related subspaces. Experimental results from two publicly available datasets show that purely individual-specific SSVEP-BCI developed by AS-TRCA further improves decoding performance. Furthermore, AS-TRCA can further improve the performance of existing calibration-based methods, including deep learning- and matrix decomposition-based methods, highlighting its potential to promote SSVEP-BCI toward practical applications.

## Figures and Tables

**Table 1:** Comparison of classification accuracy of different methods across all signal lengths.

Methods	Benchmark				Beta			
	Acc (%)	N.C	N.S	C.T (s)	Acc (%)	N.C	N.S	C.T (s)
TRCA_W	62.86 ± 18.31 ***	9	1	0.09	40.34 ± 21.70 ***	9	1	0.06
HS	59.94 ± 18.66 ***	15.5	1	357.2	39.05 ± 20.21 ***	15.0	1	220.4
NeuroXAI	59.80 ± 20.45 ***	9	1	12237	35.20 ± 22.35 ***	9	1	7974
OCLS	64.35 ± 18.77	11.5	1	1.27	42.14 ± 22.37	12.3	1	1.29
TRCA_TCT	60.86 ± 18.84 ***	9	2.5	12.02	37.17 ± 20.15 ***	9	1.6	6.49
OSS	62.15 ± 18.34	9	2.5	0.07	42.64 ± 21.16	9	4.3	0.05
TRCA	58.33 ± 19.12 ***	9	1	0.06	37.01 ± 19.96 ***	9	1	0.04
Comb	64.12 ± 18.41 ***	9	2.5	12.08	40.43 ± 21.77 ***	9	1.6	6.54
AS-TRCA	65.94 ± 18.07	11.5	2.6	1.28	43.83 ± 22.19	12.3	5.2	1.31

Acc is the averaged classification accuracy across all signal lengths (0.2 s, 0.4 s, 0.6 s, 0.8 s, and 1 s) and all subjects. N.C indicates the averaged selected optimal channel number across all subjects, N.S is the averaged optimal subspace number, and C.T is the computational time cost of training. C.T was measured on a Lenovo PC with the Intel(R) Core(TM) i5-14400F (2.50 GHz), 16 GB RAM, and 64-bit Windows 11 OS using MATLAB 2024a. C.T includes preprocessing, channel, and subspace optimization time but excludes other hyperparameter/grid search. ‘*’ is the significant difference between the proposed method and the compared method (*** *p* < 0.001). TRCA_W, BHS, NeuroXAI, and OCLS represent a channel selection performance comparison with 64 channels. TRCA_TCT and OSS represent a subspace selection performance comparison with original channels. The recognition time cost of AS-TRCA is 43.3 ± 14.8 ms at a signal length of 1 s for each test sample, while TRCA is 11.5 ± 3.7 ms.

**Table 2:** Comparison of classification accuracy of purely and existing individual-specific SSVEP-BCI.

Methods	Benchmark				BETA			
	w/o AS		w/AS		w/o AS		w/AS	
	Acc (%)	ITR	Acc (%)	ITR	Acc (%)	ITR	Acc (%)	ITR
SSCOR	49.76	133.11	56.93	149.91	33.41	67.35	41.45	87.51
TRCA	58.33	145.06	65.94	159.17	37.01	72.26	43.83	87.29
TRCA_R	61.94	155.96	68.08	170.56	44.03	93.81	51.05	107.54
msTRCA	62.24	153.46	68.83	168.74	40.66	82.36	49.45	101.70
scTRCA	63.60	162.12	68.36	173.25	47.44	105.74	51.61	114.26
TDCA	75.67	183.75	79.11	194.08	61.42	130.72	63.70	137.99
bi-SiamCA	81.44	198.22	85.12	217.67	59.49	123.02	62.27	131.09

Acc refers to classification accuracies and ITR is the information transfer rate (bit/min). All classification accuracies were averaged across all signal lengths (0.2 s, 0.4 s, 0.6 s, 0.8 s, and 1 s) and all subjects.

**Table 3:** Ablation study.

Case	Element Selection			Acc. (%)	
	OCLS	Spatial Distance Constraint	OSS	Benchmark Dataset (53.92 ± 24.26 ***)	BETA Dataset (34.20 ± 20.43 ***)
(a)	—	—	√	57.55 ± 22.91 ***	38.00 ± 21.90 ***
(b)	√	—	√	57.23 ± 23.23 ***	36.11 ± 20.43 ***
(c)	√	√	—	61.31 ± 24.90 *	39.35 ± 23.51 ***
(d)	√	√	√	63.44 ± 23.31	42.11 ± 23.32

‘*’ is the significant difference between case (d) and other cases (* *p* < 0.05, *** *p* < 0.001).

## Data Availability

The original contributions presented in this study are included in the article. Further inquiries can be directed to the corresponding author.
